# Legal-involved veterans are less likely to receive guideline-concordant colorectal cancer screening

**DOI:** 10.1186/s12913-025-12490-6

**Published:** 2025-03-04

**Authors:** Kenneth J. Nieser, Alex H. S. Harris, Ingrid A. Binswanger, Sean C. Clark, Andrea K. Finlay

**Affiliations:** 1 Center for Innovation to Implementation, VA Palo Alto Healthcare System, Palo Alto, CA USA; 2 Stanford-Surgery Policy Improvement Research and Education Center, Department of Surgery, Stanford University, Stanford, CA USA; 3 Institute for Health Research, Kaiser Permanente Colorado, Aurora, CO USA; 4 Colorado Permanente Medical Group, Denver, CO USA; 5 Division of General Internal Medicine, University of Colorado School of Medicine, Aurora, CO USA; 6 Department of Health Systems Science, Bernard J. Tyson Kaiser Permanente School of Medicine, Pasadena, CA USA; 7 Department of Veterans Affairs, Veterans Justice Programs, Washington, DC USA; 8 Department of Veterans Affairs, National Center on Homelessness Among Veterans, Menlo Park, CA USA; 9 Division of Health Systems Science, Department of Medicine, UMass Chan Medical School, Worcester, MA USA

**Keywords:** Colorectal cancer screening, Legal-involved, Veterans, Primary care

## Abstract

**Background:**

Programs to improve health care for adults with criminal legal involvement, including those who have been released from incarceration in jails or prisons or who are under court or community supervison, understandably focus on treatment for mental illness, drug overdose, and suicide. However, criminal legal-involved adults also have higher risk of developing and dying from medical conditions, such as cancer, relative to the general population. Colorectal cancer (CRC) screening among legal-involved adults, particularly those who have been incarcerated, might be delayed or missed.

**Methods:**

We conducted an observational study of national Veterans Health Administration (VHA) electronic health record data to compare the CRC screening rate between legal-involved Veterans, identified through their contact with the Veterans Justice Programs, and non-legal-involved Veterans. We included patients ages 46 to 75 eligible for average-risk screening in fiscal year 2022. Our main outcome of guideline-concordant CRC screening included stool-based testing, CT colonography, flexible sigmoidoscopy, and colonoscopy. Comparisons were estimated using an unadjusted multilevel logistic regression model with a random intercept for facility. Secondary analyses included examining associations between patient-level factors and screening receipt using adjusted models as well as assessing the variation in screening rates across 129 VHA facilities.

**Results:**

There were 27,597 legal-involved and 3,467,396 non-legal-involved patients who met screening eligibility. Only 47% of legal-involved patients were up to date with screening, compared to 54% of non-legal-involved patients (OR = 0.77 [95% CI: 0.75 to 0.79]; risk difference = -6.5% [95% CI: -7.1% to -5.9%]). Adjusted odds of screening were higher for patients with an assigned primary care provider (OR = 2.49 [95% CI: 2.48 to 2.51]). Screening rates varied widely across facilities, ranging from 24 to 75% for legal-involved patients and from 30 to 68% for non-legal-involved patients. Legal-involved patients had significantly lower screening rates at 49 facilities and a higher rate at two facilities, compared to non-legal-involved patients.

**Conclusions:**

Nearly half of VHA patients were behind on recommended CRC screening, and legal-involved VHA patients had even lower rates. Current VHA efforts to improve legal-involved patients’ connection to primary care providers may result in improved screening rates.

**Supplementary Information:**

The online version contains supplementary material available at 10.1186/s12913-025-12490-6.

## Background

Over 5.4 million adults in the United States (U.S.) were under the supervision of the criminal legal system in 2022, including almost two million incarcerated in prisons and jails and over three and a half million on probation or parole [1]. While associations between criminal legal involvement and mental health disorders, drug overdose, and suicide are well established [2–5], less is known about this population’s chronic conditions and their receipt of preventive health care [6–8]. Cancer is a leading cause of death in the aging, incarcerated population [9, 10], and formerly incarcerated adults have a higher cancer incidence compared to the general population [11]. Connecting legal-involved populations with cancer screening services could help address disparities in screen-detectable cancer prevalence, incidence, and mortality [12].

Colorectal cancer (CRC) is one of the most common types of cancer and the second leading cause of cancer death in the U.S [13]. Early stages of CRC can be detected through screening, and precancerous polyps can be removed to prevent future cancer growth [14–16]. CRC screening is a well-established, beneficial preventive health care measure that can reduce the risk of one of the leading causes of cancer death by between 20 and 70% [17]. However, many adults do not receive routine screening. Only around 60% of the eligible population between ages 45 and 75 in 2021 received guideline-concordant CRC screening, including either a colonoscopy, flexible sigmoidoscopy, stool-based test (e.g., fecal immunochemical testing), or CT colonography [18]. Legal-involved adults tend to be diagnosed with later stages of CRC compared to the general population, which suggests that screening rates in this population could be even lower [19, 20].

Studies of CRC screening rates among legal-involved adults are limited [21]. In a survey of inmates in two San Francisco jails conducted in 2002, 16 out of 55 respondents (29%) aged 50 and older reported being up to date on CRC screening [22]. In a 2010 study of administrative records from incarcerated adults in Canada awaiting sentencing or with sentences of less than two years, only 23% were up to date with CRC screening compared to about 50% of adults in the general population [23]. In general, studies of CRC screening among legal-involved adults have been hindered by a lack of screening data from jail and prison facilities and an inability to identify adults with current or former legal involvement through national surveys [24].

To address the lack of information on CRC screening among legal-involved adults, we leveraged medical record data from the Veterans Health Administration (VHA). All patients in our analysis were enrolled in the VHA, which ensured that our findings could not be explained by differences in health care coverage rates between legal-involved and non-legal-involved adults [8]. However, many VHA patients have health care coverage from multiple sources, beyond their enrollment in VHA [25, 26]. These additional forms of health insurance could differ between legal-involved and non-legal-involved patients and are a limitation of our study.

Like other legal-involved adults, legal-involved Veterans have higher rates of mental health and substance use disorders [27], and legal-involvement is associated with elevated rates of housing instability and mortality [28, 29]. Motivated by this, the VHA Homeless Programs Office implemented two programs, collectively known as the Veterans Justice Programs (VJP), aimed at connecting Veterans in the criminal legal system to necessary health and social services to curb housing instability in this population [30]. The Health Care for Reentry Veterans (HCRV) program primarily provides outreach to Veterans in prisons, and the Veterans Justice Outreach (VJO) program primarily provides outreach to Veterans in courts, jails, and other criminal legal settings. VJP outreach staff serve Veterans who have been accused of or committed all types of crimes, including violent offenses, property crimes, drug crimes, and other offenses [30, 31]. Veterans involved in less serious offenses might have spent limited or no time in incarcerated settings that would restrict their access to VHA or other community-based healthcare, whereas Veterans convicted of serious crimes, especially violent crimes, might spend years in incarcerated settings with varying levels of quality of care.

In this study, our primary aim was to estimate the unadjusted CRC screening rate among legal-involved Veterans seeking care at VHA and compare to the unadjusted rate among non-legal-involved Veterans. To identify potential drivers of screening and targets for intervention, we also estimated associations between patient-level factors and screening receipt. We were particularly interested in the relationship between having an assigned primary care provider (PCP) and screening receipt, given VJP’s current efforts to connect legal-involved Veterans with primary care. Lastly, health care quality can vary considerably across VHA facilities. Therefore, we examined variation in CRC screening and comparisons between legal-involved and non-legal-involved Veterans across VHA facilities with the intention of informing quality improvement initiatives.

## Methods

### Study design and population

Using data from the Department of Veterans Affairs (VA) Corporate Data Warehouse, we constructed a cohort of all patients across 129 VHA facilities that met criteria prescribed by the Healthcare Effectiveness Data and Information Set (HEDIS) measure for CRC screening [32]. VA’s Corporate Data Warehouse contains administrative data and electronic medical record data from all VHA facilities. The Manila VA Outpatient Clinic in the Philippines was not included in this study; only two legal-involved patients visited this facility. The Stanford University Institutional Review Board and the VA Palo Alto Research & Development Committee approved this study with a waiver of informed consent.

We included Veterans enrolled in VHA between the ages of 46 and 75 as of September 30, 2022, the last day of Fiscal Year (FY) 2022, who had an encounter within the prior three years. Following HEDIS criteria, the lower age limit allows for a one-year grace period for 45-year-old patients during FY2022. The restriction to patients with a recent encounter aligns with VHA quality measurement reporting standards for identifying patients currently or recently using VHA services as opposed to only being enrolled. Patients were excluded if they had a history of CRC or a total colectomy at any prior point; received hospice or palliative care services during FY2021-FY2022; were age 66 or older and had shown evidence of frailty and advanced illness during FY2021-FY2022; or had a life expectancy of less than 6 months. Exclusion criteria were operationalized using codes from the 2022 HEDIS Value Set. In addition, for the total colectomy and life expectancy exclusions, we identified patients using VHA-specific Health Factors following VHA quality measurement reporting.

Legal-involved VHA patients were identified as Veterans who had contact with VJP in FY2021-2022, as defined by visits to one of two clinic codes (591, 592) or an administrative record indicating contact with either of the two VJP programs. We excluded 2,739 legal-involved Veterans who did not have any encounter with a VHA facility during FY2022 because it is possible that these Veterans were still incarcerated during FY2022 and unable to receive care at a VHA facility.

### Variables

CRC screening receipt was defined by the presence of CRC screening during FY2022 or during the appropriate modality-specific lookback period. For example, given that the recommended screening interval for colonoscopy-based screening is ten years, patients were counted as screened if they had a colonoscopy in FY2022 or during the prior nine years (FY2013-FY2021). Likewise, patients with a fecal occult blood test (FOBT) or fecal immunochemical test (FIT) within one year (FY2022), a FIT-DNA within three years (FY2020-FY2022), or a flexible sigmoidoscopy or CT colonography within five years (FY2018-FY2022) were counted as up to date with their screening. Procedures were operationalized using the 2022 HEDIS Value Set with the addition of VHA-specific Health Factor data.

Sociodemographic predictors of CRC screening included age, sex, race and ethnicity, marital status, urban or rural residence, and housing instability. Race and ethnicity were self-reported and included American Indian/Alaska Native, Asian/Pacific Islander, Black/African American, Hispanic, and White. We conceptualized race and ethnicity variables as proxies for exposure to structural and interpersonal racism. Urban and rural residence was defined using U.S. Bureau of Census categories; urban areas were defined as having an urban core of at least 1,000 residents per square mile or 50,000 or more people in the urban nucleus and rural areas were defined as nonurban areas [33]. Housing instability was determined by utilization of services for homeless veterans and International Classifications of Diseases (ICD)−10 codes for housing and homelessness (Z59.0x).

Clinical predictors included VHA service-connected disability rating; assignment of a PCP; presence of a mental health disorder diagnosis in the prior two years; presence of a substance use disorder diagnosis in the prior two years; and presence of two or more medical conditions from the Charlson Comorbidity Index in the prior two years [34]. Information on the mental health disorder and substance use disorders included are provided in the Supplemental Material.

### Data analyses

We compared sociodemographic, housing instability, and clinical characteristics between legal-involved and non-legal-involved VHA patients using Pearson’s chi-squared tests. We estimated CRC screening rates for legal-involved and non-legal-involved patients in the full sample and in the subsample of patients with an assigned PCP using a multilevel logistic regression model with a random intercept for VHA facility to account for within-facility correlations. Given the expansion of U.S. Preventive Services Task Force (USPSTF) screening guidelines for CRC in 2021 to include adults between the ages of 45 and 49 [17], we also calculated screening rates without this age group to assess the sensitivity of our results to this recent update. Our main analyses are unadjusted for baseline differences between legal-involved and non-legal-involved patients. Process quality measures, such as cancer screening rates, are not risk-adjusted in general given that all patients eligible for CRC screening are expected to receive screening regardless of any baseline patient characteristics. Consequently, our comparisons should be interpreted descriptively and not causally.

In a secondary set of analyses, we examined associations between sociodemographics, housing instability, and clinical factors by fitting a multilevel logistic regression model of screening with covariates. We fit a model with covariates only for sociodemographics and housing instability as well as models that included only one predictor at a time. We created separate categories for missing responses for the race and ethnicity and marital status variables, which have missing data for 8% and 14% of patients, respectively. A missing value for one of these variables could be indicative of patients who are less likely to receive all their care at VHA; therefore, we assumed these data were missing not at random. Otherwise, models were fit on complete cases. In our initial analysis, we found significant, but modest interactions between sex and legal-involved status and between race and ethnicity and legal-involved status. Below, for simplicity, we present results from the model fit to the full sample without interaction effects. In the Supplemental Material, we provide results stratified by legal-involved status.

To examine facility-level variation in CRC screening, we estimated screening rates and 95% Wilson confidence intervals for legal-involved and non-legal-involved patients separately by VHA facility. We investigated how the association between screening receipt and legal-involved status varied across VHA facilities by fitting separate logistic regression models for each VHA facility. All analyses were performed in R version 4.4.0.

## Results

**Fig. 1:**
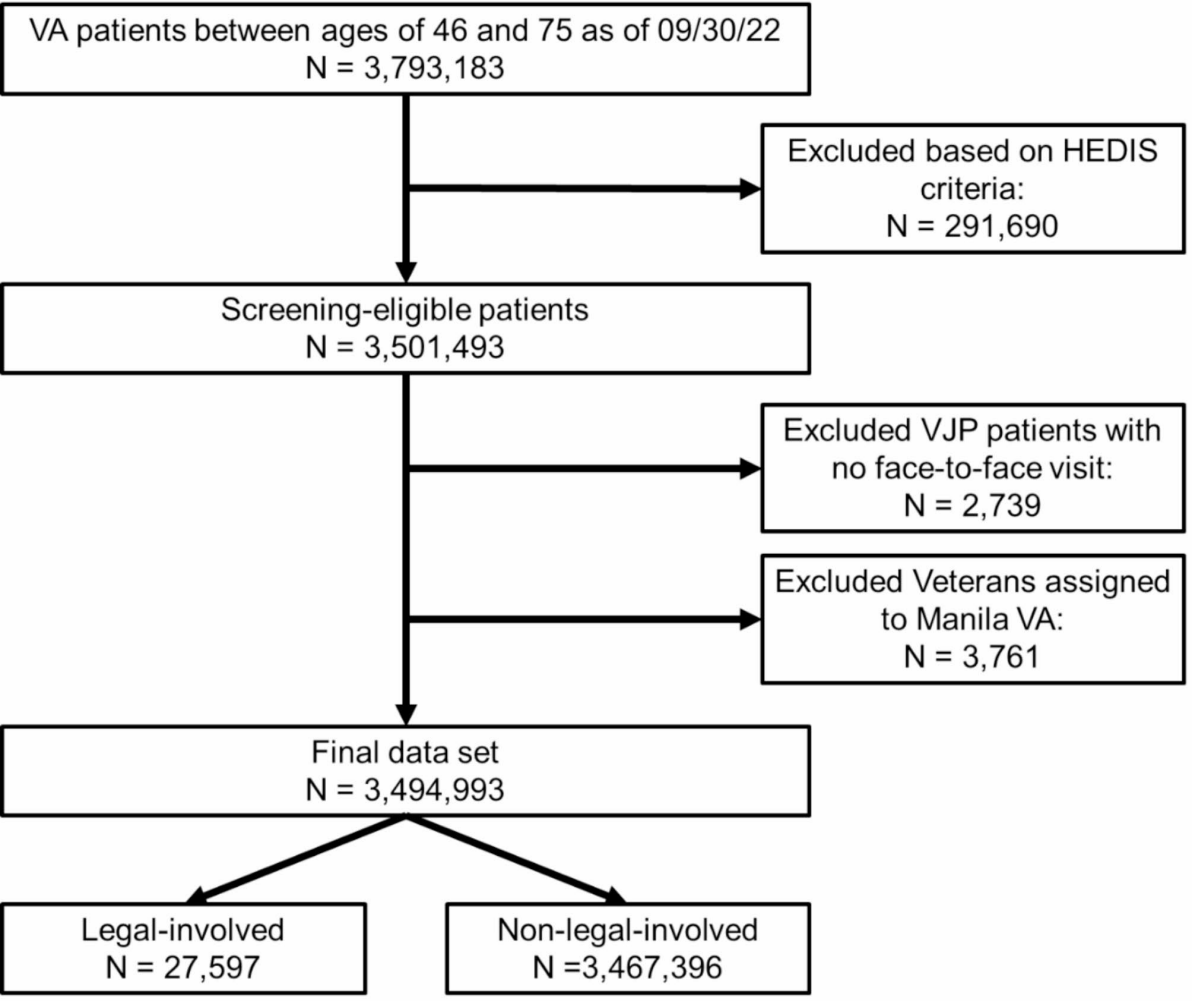
Flow diagram illustrating the number of patients included and excluded at each step of sample construction. HEDIS = Healthcare Effectiveness Data and Information Set

Our analytic data set included 27,597 legal-involved VHA patients and 3,467,396 VHA patients who are not legal-involved (Fig. 1). Of the legal-involved patients, 23,253 received outreach from the VJO program only, 3,494 from the HCRV program only, and 850 from both programs. In regression analyses, we excluded 13,205 (0.4%) patients who were missing data on sex and/or rural/urban status—140 (0.5%) legal-involved patients and 13,065 (0.4%) non-legal-involved patients. Comparisons of sociodemographics, housing instability, and clinical factors for these groups are shown in Table 1.

**Table 1:** Sociodemographics, clinical history, and screening behaviors of legal-involved and non-legal-involved veterans at VHA facilities in FY2022

	Legal-involved	Non-legal-involved		
**Overall N**	27,597	3,467,396		
**Age**	**N (%)**	**N (%)**	**RR**	***p*-value**
45–49	3,428 (12.4)	268,651 (7.7)	1.60	< 0.001
50–54	5,190 (18.8)	453,558 (13.1)	1.44	
55–59	5,125 (18.6)	483,411 (13.9)	1.33	
60–64	6,791 (24.6)	579,364 (16.7)	1.47	
65–75	7,063 (25.6)	1,682,412 (48.5)	0.53	
**Sex**				< 0.001
Female	1,911 (6.9)	358,200 (10.3)	0.67	
Male	25,686 (93.1)	3,109,101 (89.7)	1.04	
Missing	0 (0.0)	95 (0.0)	-	
**Race and ethnicity**				< 0.001
American Indian/Alaska Native	393 (1.4)	29,336 (0.8)	1.68	
Asian/Pacific Islander	344 (1.2)	69,216 (2.0)	0.62	
Black/African American	8,601 (31.2)	713,316 (20.6)	1.51	
Hispanic	1,771 (6.4)	215,591 (6.2)	1.03	
White	14,489 (52.5)	2,168,317 (62.5)	0.84	
Unknown	1,999 (7.2)	271,620 (7.8)	0.92	
**Marital status**				< 0.001
Divorced or separated	13,188 (47.8)	840,251 (24.2)	1.97	
Married	7,159 (25.9)	1,692,999 (48.8)	0.53	
Single or never married	5,828 (21.1)	354,905 (10.2)	2.06	
Widowed	857 (3.1)	80,467 (2.3)	1.34	
Unknown	565 (2.0)	498,774 (14.4)	0.14	
**Rural/Urban**				< 0.001
Rural	6,942 (25.2)	1,218,739 (35.1)	0.72	
Urban	20,515 (74.3)	2,235,664 (64.5)	1.15	
Missing	140 (0.5)	12,993 (0.4)	1.35	
**Housing instability**	11,649 (42.2)	120,392 (3.5)	12.16	< 0.001
**Service-connected disability**				< 0.001
None	11,234 (40.7)	1,500,103 (43.3)	0.94	
0–49%	4,828 (17.5)	647,413 (18.7)	0.94	
50–100%	11,535 (41.8)	1,319,880 (38.1)	1.10	
**Assigned PCP**	19,711 (71.4)	2,697,873 (77.8)	0.92	< 0.001
**Mental health disorder**	21,195 (76.8)	1,357,785 (39.2)	1.96	< 0.001
**Substance use disorder**	16,615 (60.2)	405,484 (11.7)	5.15	< 0.001
**Multiple medical conditions**	2,137 (7.7)	255,074 (7.4)	1.05	0.01

*VHA* Veterans Health Administration, *FY2022* Fiscal year from October 1, 2021 to September 30, 2022, *RR* rate ratio, *PCP* primary care provider

### CRC screening rates

Overall, 46.9% [95% CI: 46.3–47.5%] of legal-involved patients were up to date with their CRC screening compared to 53.7% [95% CI: 53.7–53.8%] of non-legal-involved patients (OR = 0.77 [95% CI: 0.75 to 0.79]; risk difference = −6.5% [95% CI: −7.1% to −5.9%]). Screening rates were lowest among the subset of legal-involved patients with prior prison incarceration (identified by HCRV contact) with a rate of 34.9% [95% CI: 33.3–36.5%] compared to patients with other forms of legal-involvement (identified by VJO contact) with a rate of 49.0% [95% CI: 48.3–49.6%] and patients with both prior prison incarceration and other forms of legal-involvement with a rate of 40.4% [95% CI: 37.1–43.7%]. Among patients with an assigned PCP, screening rates were higher for all groups compared to the full sample (6 to 7%), especially for patients enrolled in HCRV (15%, Supplemental Table 1). Screening rates among patients aged 50–75 were slightly higher (1 to 3%, Supplemental Table 1).

Colonoscopies accounted for most screenings (see Supplemental Table 2); 72.6% among legal-involved patients and 76.3% among other patients. FOBT/FIT accounted for another 24.1% among legal-involved patients and 21.1% of screenings among other patients. Formerly prison incarcerated patients were disproportionately more likely to receive screening through FOBT/FIT (31.3%) over colonoscopies (65.9%).

### Predictors of CRC screening

**Fig. 2:**
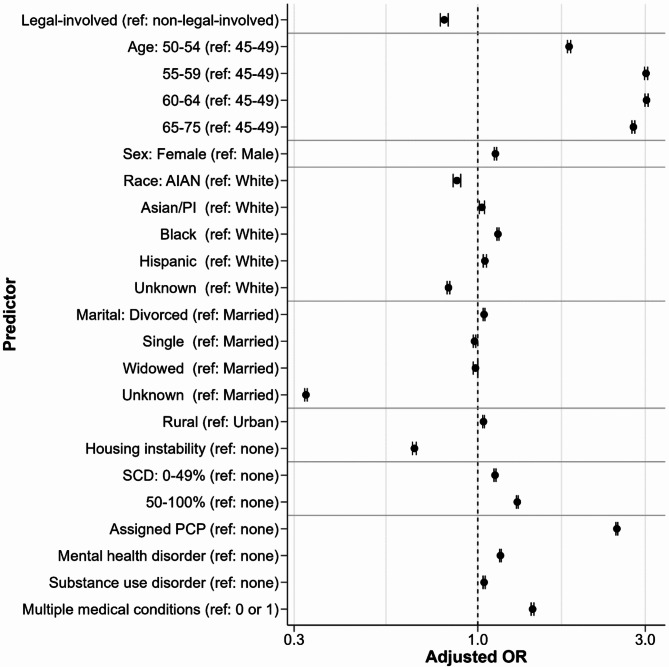
Predictors of colorectal cancer screening receipt from a multilevel logistic regression model with a random intercept for each VHA facility. Estimates are adjusted for all other covariates. Note that confidence intervals might not be visible due to their short length. VHA = Veterans Health Administration; AIAN = American Indian/Alaska Native; PI = Pacific Islander; SCD = service-connected disability rating; PCP = primary care provider

The estimated associations between legal-involved status, sociodemographics, housing instability, and clinical factors with screening receipt are displayed in Fig. 2. We report estimates from the fully adjusted model in Table 2. Results from unadjusted models and a model adjusting only for sociodemographics and housing instability variables were largely consistent with the fully adjusted model (Supplemental Table 3). After adjustment for other predictors, legal-involved status was associated with lower odds of CRC screening (OR = 0.80 [95% CI: 0.78 to 0.82]; risk difference = −4.78% [95% CI: −5.34% to −4.21%]; *p* < 0.001). Beyond legal-involved status, older age (especially being over age 55 compared to ages 45 to 49), presence of a service-connected disability, having an assigned PCP, and presence of multiple diagnosed medical conditions were associated with higher screening rates. Screening rates were slightly higher among females relative to males and among patients identifying as Black or African American relative to White. Screening rates were lower among American Indian or Alaska Native patients and patients of unknown race and ethnicity compared to White patients, patients of unknown marital status compared to married patients, and among patients with housing instability. In our stratified analyses (Supplemental Tables 4 and 5 and Supplemental Fig. 1), associations were mostly consistent across legal-involved and non-legal-involved Veterans.

**Table 2:** Predictors of colorectal cancer screening

Predictor	Adjusted risk difference (95% CI)^b^	Adjusted OR (95% CI)^c^	*p*-value^c^
**Legal-involved (ref: non-legal-involved)**	−4.78 (−5.34, −4.21)	0.80 (0.78, 0.82)	< 0.001
**Age**			
45–49	ref	ref	ref
50–54	12.98 (12.75, 13.21)	1.82 (1.80, 1.84)	< 0.001
55–59	24.09 (23.87, 24.31)	3.02 (2.98, 3.05)	< 0.001
60–64	24.16 (23.94, 24.38)	3.03 (2.99, 3.06)	< 0.001
65–75	22.30 (22.10, 22.50)	2.78 (2.75, 2.80)	< 0.001
**Female (ref: Male)**	2.49 (2.32, 2.65)	1.12 (1.11, 1.13)	< 0.001
**Race and ethnicity**			
American Indian/Alaska Native	−2.97 (−3.52, −2.43)	0.87 (0.85, 0.89)	< 0.001
Asian/Pacific Islander	0.59 (0.22, 0.96)	1.03 (1.01, 1.05)	0.002
Black/African American	2.86 (2.72, 2.99)	1.14 (1.13, 1.15)	< 0.001
Hispanic	1.02 (0.79, 1.24)	1.05 (1.04, 1.06)	< 0.001
White	ref	ref	ref
Unknown	−4.19 (−4.39, −3.99)	0.83 (0.82, 0.83)	< 0.001
**Marital status**			
Divorced or separated	0.94 (0.81, 1.06)	1.04 (1.04, 1.05)	0.01
Married	ref	ref	ref
Single or never married	−0.47 (−0.65, −0.29)	0.98 (0.97, 0.99)	< 0.001
Widowed	−0.33 (−0.67, 0.00)	0.99 (0.97, 1.00)	0.05
Unknown	−25.13 (−25.33, −24.92)	0.32 (0.32, 0.33)	< 0.001
**Rural (ref: Urban)**	0.81 (0.70, 0.93)	1.04 (1.03, 1.04)	< 0.001
**Housing instability**	−9.06 (−9.33, −8.79)	0.66 (0.65, 0.67)	< 0.001
**Service-connected disability**			
None	ref	ref	ref
0–49%	2.49 (2.34, 2.63)	1.12 (1.11, 1.13)	< 0.001
50–100%	5.64 (5.51, 5.77)	1.30 (1.29, 1.30)	< 0.001
**Assigned PCP (ref: none)**^a^	20.66 (20.53, 20.80)	2.49 (2.48, 2.51)	< 0.001
**Mental health disorder (ref: none)**	3.21 (3.10, 3.33)	1.16 (1.15, 1.17)	< 0.001
**Substance use disorder (ref: none)**	0.90 (0.74, 1.06)	1.04 (1.03, 1.05)	< 0.001
**Multiple medical conditions (ref: 0 or 1)**	7.76 (7.57, 7.96)	1.43 (1.42, 1.45)	< 0.001

^a^*PCP * primary care provider

^b^Estimates of average marginal effects calculated from a multilevel logistic regression model with random intercepts for facility and adjusted for all covariates in the table; confidence intervals from delta method approximation. Estimates are presented as percentage point differences

^c^Estimates from the adjusted model; confidence intervals and *p*-values from Wald tests

### Facility-level analyses

**Fig. 3:**
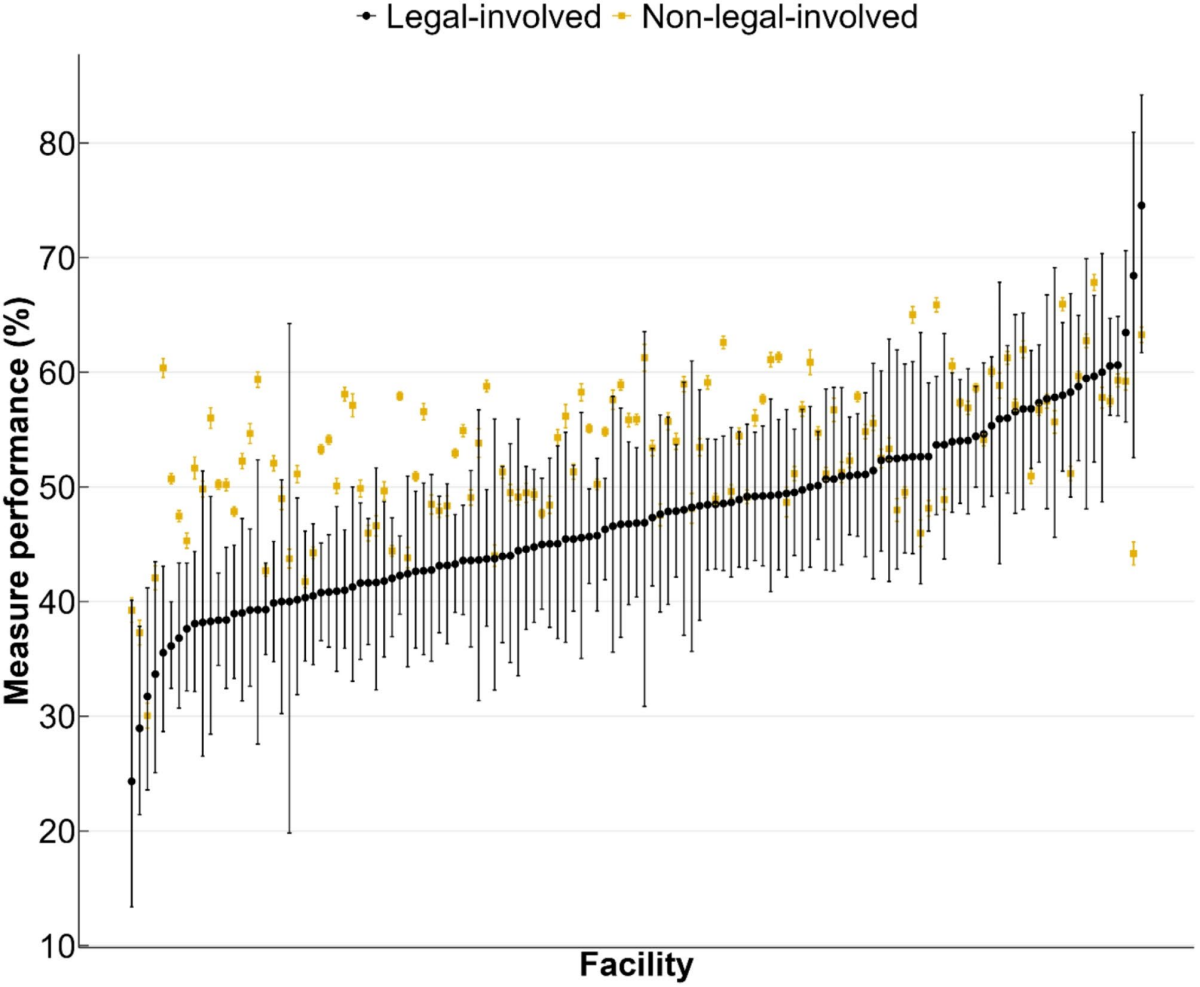
Colorectal cancer screening rates by VHA facility for legal-involved (black dot) and non-legal-involved patients (yellow square) in FY2022 (October 1, 2021 to September 30). Facility-specific estimates are ordered by the facility-specific estimate among legal-involved patients

Across VHA facilities, screening rates ranged from 24.3 to 74.5% for legal-involved patients and from 30.0 to 67.8% for non-legal-involved patients (Fig. 3). The screening rate among legal-involved patients was significantly lower compared to non-legal-involved patients at 49 facilities and higher at two facilities (Supplemental Fig. 2).

## Discussion

Legal-involved adults have higher rates of cancer mortality, yet few studies of cancer screening rates among this population exist [21]. Most of the prior studies on this topic focused on cervical cancer screening, and studies on CRC screening have been limited to self-report data, a subset of the possible screening modalities, and/or small sample sizes [21]. While national surveys, such as the National Health Interview Survey and National Health and Nutrition Examination Survey, generally provide key sources of information on cancer screening rates, measures for identifying adults with current or prior legal-involvement are often absent and incarcerated populations are typically excluded [24]. Our use of national electronic health record data and well-established, standardized quality measurement criteria for guideline-concordant CRC screening to estimate CRC screening rates among legal-involved patients fills a major gap in knowledge on screening rates among this population [12, 21].

In line with prior studies that have suggested poor CRC screening rates among legal-involved adults [21], legal-involved VHA patients were less likely to be up to date with CRC screening relative to non-legal-involved VHA patients. The association between legal-involved status and screening was largely unchanged after controlling for sociodemographics, housing instability, and clinical factors. In comparison to other factors, such as age, housing instability, having an assigned PCP, and having multiple medical conditions, legal-involved status had a more moderate association with screening. Nonetheless, these findings suggest that continued efforts are needed to address preventive health needs, such as CRC screening, of legal-involved adults.

Several factors could explain the lower CRC screening rate among legal-involved patients. Prisons and jails often lack the capacity to provide preventive care, and many leave incarceration without adequate transition to basic preventive health care. Colonoscopies, which account for a majority of screenings, require significant patient effort and resources, including bowel preparation, taking time off work, and obtaining transportation, which might present barriers for legal-involved adults who often are disadvantaged socioeconomically and likely have other competing and stressful demands. Legal-involved adults can have difficulty securing housing and employment and reconnecting with family and friends, which could affect their capacity to prioritize important preventive health behaviors [35]. Patients might also encounter discrimination from health care workers regarding their criminal legal-involved status [36]. In addition, the invasiveness and vulnerability of receiving a colonoscopy might evoke experiences with strip frisking, or men might view it as a violation of their masculinity [37]. Rhode Island departments of public health and corrections provide one model for how to improve CRC screening using FIT for incarcerated adults, but concerns were also observed in this patient population [38].

While CRC incidence has been declining over the last few decades, incidence among adults younger than 50 has been increasing at a swift pace [18, 39]. In light of the updated USPSTF guidance in May 2021 to screen average-risk, asymptomatic adults between the ages of 45–49 [17], estimating the CRC screening rate among legal-involved adults, who tend to be younger than the general population, is timely. Excluding the newly eligible 45- to 49-year-olds from our analysis resulted in higher screening rates, indicating that this younger group has a lower screening rate for both legal-involved and non-legal-involved patients, which is consistent with national survey data among the general population [18]. Reduced screening rates among this younger cohort could partly be due to a lack of clear messaging of the recently updated guidelines or differences in perceived risk [40, 41].

Patients with an assigned PCP were more likely to receive guideline-concordant screening, which is also supported by previous studies [42]. This supports the importance of VJP’s current efforts to connect legal-involved adults with primary care in improving CRC screening rates. Once patients are engaged with primary care, several strategies have been effective at supporting primary care practices to increase screening, including academic detailing, practice facilitation, and patient incentive programs, supplemented by patient navigation and patient reminders [43, 44].

Organized screening programs that include automatic mailing of FIT kits to eligible patients are another promising strategy for increasing CRC screening rates [45, 46]. In 2021, VHA began piloting its own mailed FIT program at several facilities and is in the process of expanding across the system [47]. Early reports have suggested that FIT return rates can be low (33–35%), and current research is investigating strategies to encourage patients to return FIT kits, such as automated reminders [47, 48]. Timely follow-up colonoscopies for patients with positive FIT are an important aspect and challenge with stool-based screening [49, 50]. The comparative effectiveness of FIT and screening colonoscopy is currently under investigation through several large randomized controlled trials [51–53].

We found a wide range in screening rates across VHA facilities for both legal-involved and non-legal-involved patients, and we identified 49 facilities where legal-involved patients had significantly lower screening rates. Differences in screening rates across VHA facilities might reflect varying health care priorities or resources across the VHA’s system of 18 regional integrated service networks. Variation might also be explained by differences in patient health behaviors and attitudes toward screening. Further research will be needed to understand what is driving this variability to inform quality improvement efforts.

### Strengths and limitations

Major strengths of our study are the identification of legal-involved patients through their participation in VJP and use of national VHA electronic medical record data. Prior studies have had small sample sizes, were specific to certain regions, or lacked screening data [19, 21–23]. In addition, our study estimated CRC screening rates according to HEDIS criteria rather than self-reported data from national health surveys [54]. Our study included a broad range of VHA patients with an encounter in the last three years, which might explain the discrepancy between our estimated rates for non-legal-involved patients and the higher rates previously reported (77–80%) that were based on manual chart review of higher utilizing patients [55].

Given that our study was limited to VHA patients, differences in health care eligibility are unlikely to explain the differences in screening rates between legal-involved and non-legal-involved patients. However, non-legal-involved patients might be more likely to have additional forms of health insurance outside of VHA, which could be a potential explanation for the screening rate differences.

Several limitations of our study exist. First, some legal-involved patients might be misclassified. We identified legal-involved Veterans according to contact with VJP; however, not all legal-involved Veterans receive VJP services. Second, patients who received CRC screening might be misclassified. Veterans have multiple forms of health insurance, and health care services that Veterans received outside of VHA facilities generally were not captured in our dataset. VHA providers can record CRC screening events that occur outside the VHA in VHA’s electronic health record, which might lessen the impact of this limitation. However, we were unable to quantify the extent to which CRC screening was documented via this mechanism. Third, the COVID-19 pandemic impacted patients’ utilization of preventive health care, which likely led to lower-than-average screening rates during FY2022.

## Conclusions

Almost half of VHA patients were not up to date on their screening, and rates were approximately 7 percentage points lower among legal-involved patients. Quality improvement efforts could prioritize patients between ages 45 and 55 and VHA facilities with the largest discrepancies between legal-involved and non-legal-involved patients. In addition, the growth of the mailed FIT program and current VHA Homeless Programs Office efforts to connect legal-involved patients with primary care are promising strategies to increase preventive health behaviors in this population.

## Supplementary Information

Supplementary Material 1.

## Data Availability

The data set generated and analyzed during the study is not publicly available. The United States Department of Veterans Affairs (VA) places legal restrictions on access to Veterans’ health care data, which includes both identifying data and sensitive patient information.

## Abbreviations

CRC: Colorectal cancer
FIT: Fecal immunochemical test
FOBT: Fecal occult blood test
FY: Fiscal year
HCRV: Health Care for Reentry Veterans
HEDIS: Healthcare Effectiveness Data and Information Set
ICD: International Classification of Diseases
PCP: Primary care provider
U.S.: United States
USPSTF: U.S. Preventive Services Task Force
VHA: Veterans Health Administration
VJO: Veterans Justice Outreach
VJP: Veterans Justice Programs
