# Supplementary material for: Legal-involved veterans are less likely to receive guideline-concordant colorectal cancer screening
Source: BMC Health Serv Res. 2025 Mar 4;25:333. doi: 10.1186/s12913-025-12490-6 (PMC11877804; doi:10.1186/s12913-025-12490-6)

Supplemental Material

# Additional detail on variable definitions

Homeless status was determined by utilizing services for homeless veterans (clinic codes: 504,511,501,507,508,515,522,528,529,530,555,556,590; bed section codes: 28, 29, 37, 39) or International Classifications of Diseases (ICD)-10th Edition CM codes for housing and homelessness (Z59.0x).

Legal-involved status was defined by clinic codes (591, 592) or an administrative record indicating contact with either of the Veterans Justice Programs. Administrative records were pulled from the Homeless Operations Management and Evaluation System (HOMES), which is the data collection system for all the programs within the VHA Homeless Programs Office.

# Screening rates sensitivity analyses

**Supplemental Table 1: Colorectal cancer screening rates and 95% confidence intervals for legal-involved and non-legal-involved Veterans in FY2022.**

| **Group** | **Ages 45-75** | **Ages 50-75** | **Ages 45-75 & assigned PCP** | **Ages 50-75 & assigned PCP** |
| --- | --- | --- | --- | --- |
| Legal-involved | 46.9%  (46.3%, 47.5%) | 49.4%  (48.8%, 50.0%) | 53.1%  (52.5%, 53.8%) | 55.7%  (55.0%, 56.5%) |
| VJO only | 49.0%  (48.3%, 49.6%) | 51.8%  (51.1%, 52.5%) | 53.5%  (52.8%, 54.3%) | 56.2%  (55.4%, 57.0%) |
| HCRV only | 34.9%  (33.3%, 36.5%) | 36.1%  (34.4%, 37.7%) | 50.9%  (48.3%, 53.4%) | 52.5%  (49.9%, 55.1%) |
| VJO + HCRV | 40.4%  (37.1%, 43.7%) | 42.3%  (38.9%, 45.9%) | 46.4%  (42.0%, 50.8%) | 48.6%  (43.9%, 53.3%) |
| Non-legal-involved | 53.7%  (53.7%, 53.8%) | 55.5%  (55.4%, 55.5%) | 60.6%  (60.5%, 60.7%) | 62.4%  (62.3%, 62.4%) |

FY2022 = Fiscal year from October 1, 2021 to September 30; VJO = Veteran Justice Outreach; HCRV = Health Care for Re-entry Veterans

# Screening modality by legal-involved status and VJP group

**Supplemental Table 2: Distribution of types of CRC screening received by legal-involved status and VJP group. This table includes only patients who met the screening guidelines.**

| **Screening modality** | **Legal-involved** | **VJO only** | **HCRV only** | **VJO + HCRV** | **Non-legal-involved** |
| --- | --- | --- | --- | --- | --- |
| Colonoscopy | 72.6% | 73.7% | 65.9% | 62.4% | 76.3% |
| FOBT/FIT | 24.1% | 23.1% | 31.3% | 32.1% | 21.1% |
| FIT-DNA | 1.5% | 1.4% | 1.6% | 2.6% | 1.5% |
| Flexible sigmoidoscopy | 1.6% | 1.7% | 1.1% | 2.3% | 0.8% |
| CT colonography | 0.1% | 0.1% | 0.0% | 0.6% | 0.2% |

# Additional results from multilevel logistic regression model of screening receipt

**Supplemental Table 3: Model results for the full sample**

The table below shows OR estimates and 95% Wald CIs associated with parameters from multilevel logistic regression models of CRC screening receipt among legal-involved Veterans. In Model 0, we fit each predictor separately to obtain unadjusted estimates. Model 1 includes only sociodemographic predictors. Model 2 includes all sociodemographic and clinical predictors.

|  | **Model 0** | **Model 1** | **Model 2** |
| --- | --- | --- | --- |
| **Predictor** | **OR (95% CI)** | **OR (95% CI)** | **OR (95% CI)** |
| **Legal-involved**  **(ref: non-legal-involved)** | 0.77 (0.75, 0.79) | 0.80 (0.78, 0.82) | 0.80 (0.78, 0.82) |
| **Age** |  |  |  |
| 45-49 | ref | ref | ref |
| 50-54 | 1.76 (1.75, 1.78) | 1.78 (1.77, 1.80) | 1.82 (1.80, 1.84) |
| 55-59 | 2.81 (2.78, 2.84) | 2.87 (2.85, 2.90) | 3.02 (2.98, 3.05) |
| 60-64 | 2.84 (2.81, 2.87) | 2.84 (2.82, 2.87) | 3.03 (2.99, 3.06) |
| 65-75 | 2.69 (2.66, 2.71) | 2.66 (2.63, 2.68) | 2.78 (2.75, 2.80) |
| **Female (ref: Male)** | 1.08 (1.08, 1.09) | 1.14 (1.14, 1.15) | 1.12 (1.11, 1.13) |
| **Race and ethnicity** |  |  |  |
| American Indian or Alaska Native | 0.83 (0.81, 0.85) | 0.88 (0.86, 0.90) | 0.87 (0.85, 0.89) |
| Asian or Pacific Islander | 1.01 (1.00, 1.03) | 1.06 (1.05, 1.08) | 1.03 (1.01, 1.05) |
| Black or African American | 1.21 (1.21, 1.22) | 1.21 (1.21, 1.22) | 1.14 (1.13, 1.15) |
| Hispanic | 1.01 (1.00, 1.02) | 1.07 (1.06, 1.08) | 1.05 (1.04, 1.06) |
| White | ref | ref | ref |
| Unknown | 0.61 (0.61, 0.62) | 0.77 (0.76, 0.78) | 0.83 (0.82, 0.83) |
| **Marital status** |  |  |  |
| Divorced or separated | 1.03 (1.02, 1.03) | 1.03 (1.02, 1.03) | 1.04 (1.04, 1.05) |
| Married | ref | ref | ref |
| Single or never Married | 0.90 (0.89, 0.90) | 0.95 (0.95, 0.96) | 0.98 (0.97, 0.99) |
| Widowed | 1.04 (1.02, 1.05) | 0.96 (0.94, 0.97) | 0.99 (0.97, 1.00) |
| Unknown | 0.19 (0.19, 0.19) | 0.19 (0.19, 0.20) | 0.32 (0.32, 0.33) |
| **Rural (ref: Urban)** | 1.04 (1.04, 1.05) | 1.04 (1.03, 1.04) | 1.04 (1.03, 1.04) |
| **Housing instability** | 0.79 (0.78, 0.80) | 0.62 (0.62, 0.63) | 0.66 (0.65, 0.67) |
| **Service-connected disability** |  |  |  |
| None | ref |  | ref |
| 0-49% | 1.54 (1.53, 1.55) |  | 1.12 (1.11, 1.13) |
| 50-100% | 1.89 (1.88, 1.90) |  | 1.30 (1.29, 1.30) |
| **Assigned PCP (ref: none)** | 3.66 (3.64, 3.68) |  | 2.49 (2.48, 2.51) |
| **Mental health disorder (ref: none)** | 1.50 (1.49, 1.50) |  | 1.16 (1.15, 1.17) |
| **Substance use disorder (ref: none)** | 1.23 (1.22, 1.24) |  | 1.04 (1.03, 1.05) |
| **Multiple medical conditions (ref: 0 or 1)** | 1.95 (1.94, 1.97) |  | 1.43 (1.42, 1.45) |

PCP = primary care provider

**Supplemental Table 4: Model results for legal-involved Veterans**

The table below shows OR estimates and 95% Wald CIs associated with parameters from multilevel logistic regression models of CRC screening receipt among legal-involved Veterans. In Model 0, we fit each predictor separately to obtain unadjusted estimates. Model 1 includes only sociodemographic predictors. Model 2 includes all sociodemographic and clinical predictors.

|  | **Model 0** | **Model 1** | **Model 2** |
| --- | --- | --- | --- |
| **Predictor** | **OR (95% CI)** | **OR (95% CI)** | **OR (95% CI)** |
| **Age** |  |  |  |
| 45-49 | ref | ref | ref |
| 50-54 | 1.58 (1.44, 1.73) | 1.58 (1.44, 1.74) | 1.61 (1.46, 1.77) |
| 55-59 | 2.46 (2.24, 2.70) | 2.52 (2.29, 2.76) | 2.69 (2.44, 2.96) |
| 60-64 | 2.58 (2.36, 2.82) | 2.65 (2.42, 2.90) | 2.95 (2.69, 3.24) |
| 65-75 | 2.71 (2.48, 2.95) | 2.75 (2.51, 3.00) | 3.13 (2.85, 3.44) |
| **Female (ref: Male)** | 1.29 (1.17, 1.41) | 1.46 (1.32, 1.61) | 1.29 (1.16, 1.42) |
| **Race and ethnicity** |  |  |  |
| American Indian or Alaska Native | 0.67 (0.54, 0.82) | 0.69 (0.56, 0.86) | 0.69 (0.56, 0.86) |
| Asian or Pacific Islander | 0.94 (0.75, 1.17) | 0.97 (0.78, 1.22) | 0.94 (0.75, 1.19) |
| Black or African American | 1.29 (1.22, 1.36) | 1.23 (1.16, 1.31) | 1.15 (1.08, 1.22) |
| Hispanic | 1.04 (0.94, 1.16) | 1.11 (0.99, 1.23) | 1.05 (0.94, 1.17) |
| White | ref | ref | ref |
| Unknown | 0.68 (0.62, 0.75) | 0.73 (0.66, 0.80) | 0.80 (0.72, 0.89) |
| **Marital status** |  |  |  |
| Divorced or separated | 0.89 (0.84, 0.95) | 0.84 (0.79, 0.90) | 0.92 (0.87, 0.98) |
| Married | ref | ref | ref |
| Single or never Married | 0.78 (0.73, 0.84) | 0.76 (0.70, 0.82) | 0.84 (0.78, 0.91) |
| Widowed | 1.00 (0.87, 1.16) | 0.82 (0.71, 0.95) | 0.91 (0.79, 1.06) |
| Unknown | 0.36 (0.30, 0.44) | 0.40 (0.33, 0.49) | 0.51 (0.41, 0.62) |
| **Rural (ref: Urban)** | 0.92 (0.87, 0.97) | 0.94 (0.89, 1.00) | 0.96 (0.91, 1.03) |
| **Housing instability** | 0.91 (0.87, 0.96) | 0.87 (0.83, 0.92) | 0.88 (0.83, 0.93) |
| **Service-connected disability** |  |  |  |
| None | ref |  | ref |
| 0-49% | 1.32 (1.24, 1.42) |  | 1.31 (1.22, 1.41) |
| 50-100% | 1.66 (1.57, 1.75) |  | 1.58 (1.49, 1.68) |
| **Assigned PCP (ref: none)** | 2.44 (2.31, 2.58) |  | 2.01 (1.89, 2.13) |
| **Mental health disorder (ref: none)** | 1.80 (1.70, 1.91) |  | 1.48 (1.38, 1.58) |
| **Substance use disorder (ref: none)** | 1.19 (1.13, 1.25) |  | 1.03 (0.97, 1.09) |
| **Multiple medical conditions (ref: 0 or 1)** | 2.10 (1.92, 2.31) |  | 1.67 (1.51, 1.83) |

PCP = primary care provider

**Supplemental Table 5: Model results for non-legal-involved Veterans**

The table below shows OR estimates and 95% Wald CIs associated with parameters from multilevel logistic regression models of CRC screening adherence among non-legal-involved Veterans. In Model 0, we fit each predictor separately to obtain unadjusted estimates. Model 1 includes only sociodemographic predictors. Model 2 includes all sociodemographic and clinical predictors.

|  | **Model 0** | **Model 1** | **Model 2** |
| --- | --- | --- | --- |
| **Predictor** | **OR (95% CI)** | **OR (95% CI)** | **OR (95% CI)** |
| **Age** |  |  |  |
| 45-49 | ref | ref | ref |
| 50-54 | 1.77 (1.75, 1.78) | 1.79 (1.77, 1.81) | 1.82 (1.80, 1.84) |
| 55-59 | 2.81 (2.78, 2.84) | 2.88 (2.85, 2.91) | 3.02 (2.99, 3.05) |
| 60-64 | 2.84 (2.81, 2.87) | 2.85 (2.82, 2.88) | 3.03 (3.00, 3.06) |
| 65-75 | 2.68 (2.66, 2.71) | 2.66 (2.63, 2.68) | 2.77 (2.75, 2.80) |
| **Female (ref: Male)** | 1.08 (1.07, 1.09) | 1.14 (1.13, 1.15) | 1.12 (1.11, 1.13) |
| **Race and ethnicity** |  |  |  |
| American Indian or Alaska Native | 0.84 (0.82, 0.86) | 0.88 (0.86, 0.91) | 0.88 (0.85, 0.90) |
| Asian or Pacific Islander | 1.01 (1.00, 1.03) | 1.07 (1.05, 1.08) | 1.03 (1.01, 1.05) |
| Black or African American | 1.22 (1.21, 1.22) | 1.21 (1.21, 1.22) | 1.14 (1.13, 1.15) |
| Hispanic | 1.01 (1.00, 1.02) | 1.07 (1.06, 1.08) | 1.05 (1.04, 1.06) |
| White | ref | ref | ref |
| Unknown | 0.61 (0.61, 0.62) | 0.77 (0.76, 0.78) | 0.83 (0.82, 0.83) |
| **Marital status** |  |  |  |
| Divorced or separated | 1.03 (1.03, 1.04) | 1.03 (1.03, 1.04) | 1.04 (1.04, 1.05) |
| Married | ref | ref | ref |
| Single or never married | 0.90 (0.90, 0.91) | 0.96 (0.95, 0.96) | 0.98 (0.97, 0.99) |
| Widowed | 1.04 (1.03, 1.06) | 0.96 (0.95, 0.97) | 0.99 (0.97, 1.00) |
| Unknown | 0.19 (0.19, 0.19) | 0.19 (0.19, 0.20) | 0.32 (0.32, 0.33) |
| **Rural (ref: Urban)** | 1.04 (1.03, 1.04) | 1.04 (1.03, 1.04) | 1.04 (1.03, 1.04) |
| **Housing instability** | 0.80 (0.79, 0.81) | 0.61 (0.60, 0.62) | 0.65 (0.64, 0.66) |
| **Service-connected disability** |  |  |  |
| None | ref |  | ref |
| 0-49% | 1.54 (1.54, 1.55) |  | 1.12 (1.11, 1.13) |
| 50-100% | 1.90 (1.89, 1.91) |  | 1.29 (1.29, 1.30) |
| **Assigned PCP (ref: none)** | 3.67 (3.65, 3.69) |  | 2.49 (2.48, 2.51) |
| **Mental health disorder (ref: none)** | 1.50 (1.50, 1.51) |  | 1.16 (1.15, 1.17) |
| **Substance use disorder (ref: none)** | 1.25 (1.24, 1.26) |  | 1.04 (1.04, 1.05) |
| **Multiple medical conditions (ref: 0 or 1)** | 1.95 (1.93, 1.97) |  | 1.43 (1.42, 1.44) |

PCP = primary care provider

**Supplemental Figure 1: Plot of adjusted ORs from models stratified by legal-involved status**


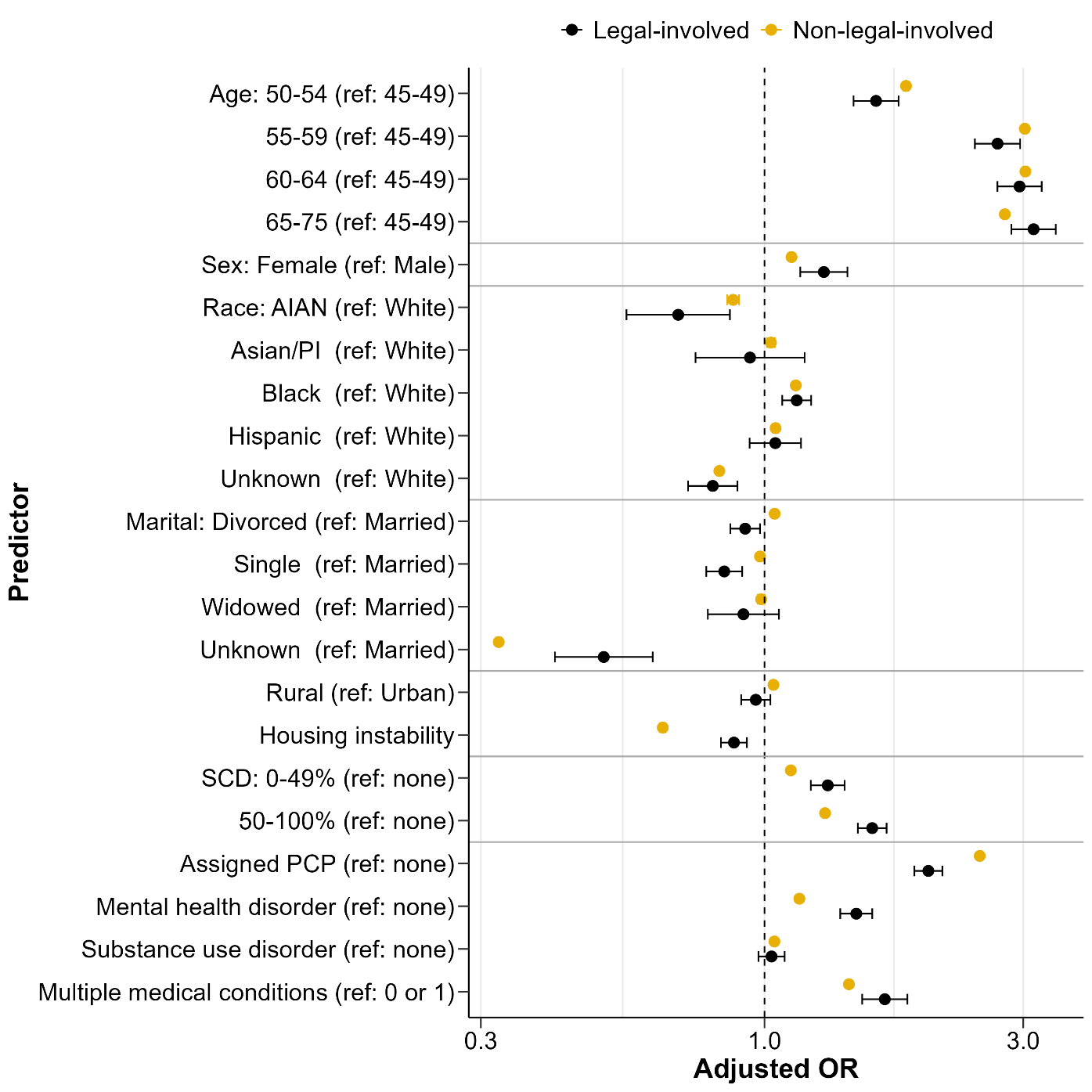


Predictors of colorectal cancer screening, stratified by legal-involved status. Note that confidence intervals for non-legal-involved Veterans might not be visible due to their short length.

AIAN = American Indian/Alaska Native; PI = Pacific Islander; SCD = service-connected disability rating; PCP = primary care provider.

# Results from facility-specific models of screening receipt

**Supplemental Figure 2: Facility-specific ORs of colorectal cancer screening among legal-involved Veterans compared to non-legal-involved Veterans.**


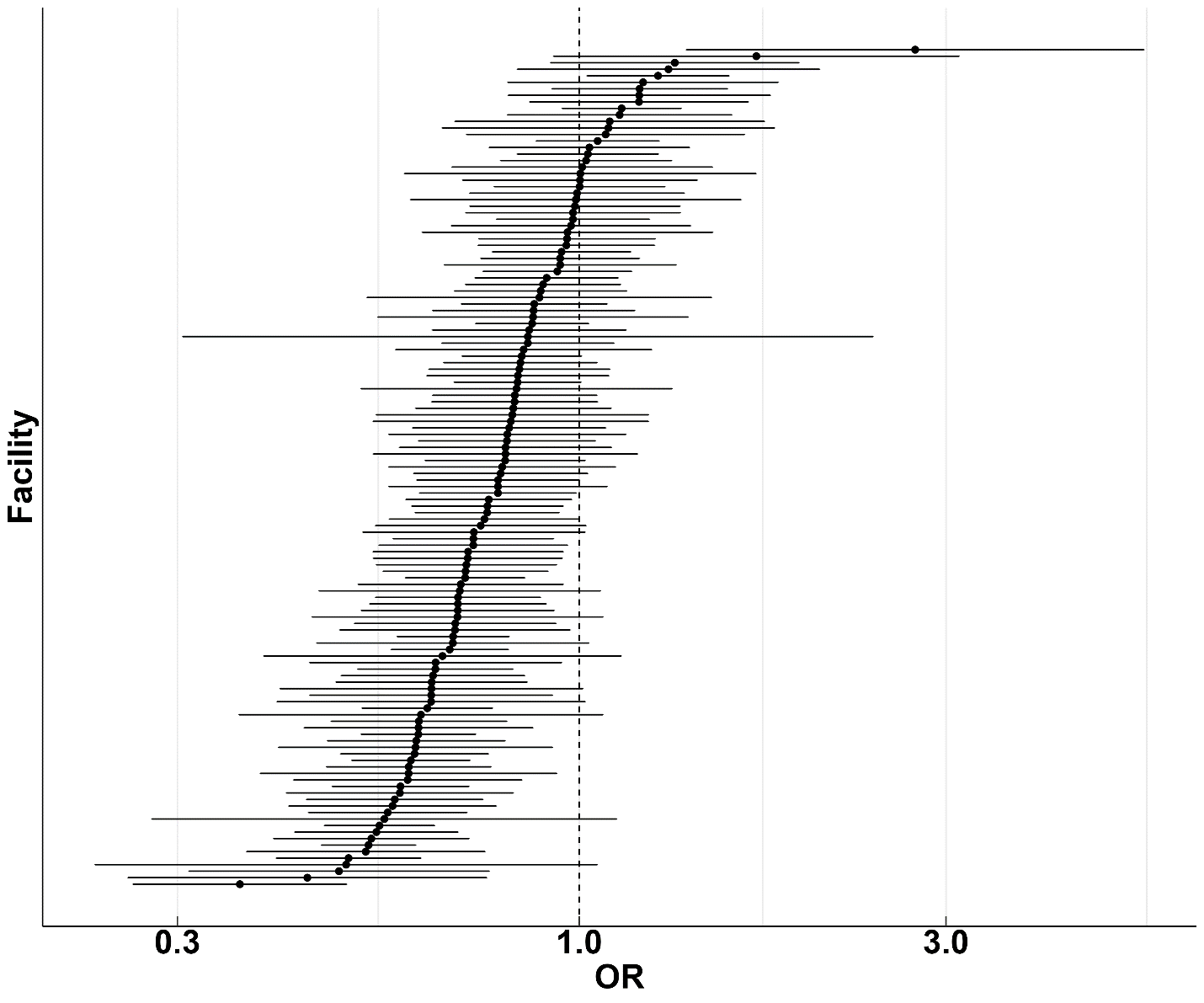

Supplement: Supplementary file 1 — Supplementary Material 1. [file 12913_2025_12490_MOESM1_ESM.docx]
